# Photography and radical psychiatry in Italy in the 1960s. The case of the photobook *Morire di Classe* (1969)

**DOI:** 10.1177/0957154X14550136

**Published:** 2015-03

**Authors:** John Foot

**Affiliations:** University of Bristol

**Keywords:** Antipsychiatry, Franco Basaglia, Italy, photography, psychiatric reforms

## Abstract

In the 1960s Franco Basaglia, the Director of a Psychiatric Hospital in a small city on the edge of Italy (Gorizia), began to transform that institution from the inside. He introduced patient meetings and set up a kind of Therapeutic Community. In 1968 he asked two photographers – Carla Cerati and Gianni Berengo Gardin – to take photos inside Gorizia and other asylums. These images were then used in a photobook called *Morire di Classe* (To Die Because of your Class) (1969). This article re-examines in detail the content of this celebrated book and its history, and its impact on the struggle to reform and abolish large-scale psychiatric institutions. It also places the book in its social and political context and as a key text of the anti-psychiatry movement of the 1960s.

‘Power … is most effective when invisible.’ (Gittins, 1998: 46)

The first time I saw a psychiatric hospital on the inside was in Gorizia under Franco Basaglia’s Directorship – where there were no longer any strait-jackets, but there was still a sense of poverty and people who had been inside for fifty years or so who no longer noticed the presence of walls or bars. Basaglia told me that they were so institutionalized, that when they went out for walks they would turn back at the point where the wall used to be. Their lives had been destroyed by the hospital. (Carla Cerati)^1^

In the 1960s Franco Basaglia, the Director of a psychiatric hospital in Gorizia, a small city on the north-eastern edge of Italy, began to transform that institution from the inside. He introduced patient-led meetings and set up a kind of Therapeutic Community. Many patients were free to come and go almost as they pleased, and gates, walls and fences were pulled down (often by the patients themselves, in set-piece ‘happenings’). By 1967 all the wards were unlocked. Basaglia, working with his wife Franca and a small but highly motivated team (which was known as an *équipe*), argued strongly for the abolition of large-scale psychiatric institutions, which he often compared to concentration camps or prisons. The Gorizia experiment became famous towards the end of the 1960s through the publication of a best-selling collective book *L’istituzione negata* (The Institution Denied; Basaglia, 1968) and a TV documentary shot inside the asylum that was seen by millions across Italy (*I Giardini di Abele*, Zavoli, 1969).^2^ A powerful movement emerged around this time that called for the reform of psychiatric theories and practices. It used film, photography, texts and other means to get its message across.^3^

This article will focus on one aspect of Basaglia’s struggle against what he called, after Goffman, ‘total institutions’.^4^ In 1968 Basaglia asked two photographers – Carla Cerati and Gianni Berengo Gardin – to take photographs inside Gorizia and other asylums. These images were then used in a photobook published in 1969 called *Morire di Classe*, which roughly translates as ‘To die because of your class’.^5^

This article re-examines the history of this celebrated book (both before and after publication) and its content in detail, as well its impact on the struggle to reform and abolish large-scale psychiatric institutions. We will also look at the reputation of this volume. Big claims have been made for the book and its effects on Italians – but little evidence has been provided to back up these claims. Moreover, few of those who have looked at *Morire di Classe* have analysed in detail the layout and text of the book itself – and the relationship between *Morire di Classe* and other texts produced by Basaglia and the *équipe*. The one exception to this is David Forgacs’ 2014 book *Italy’s Margins*, which is a key reference point for this piece.^6^

## Introduction: a radical photobook

It had a striking lilac cover and a bold title, and it was not like a ‘normal book’: in size, in shape, and inside, in terms of its content. Its images screamed out at you, sometimes almost literally, from the pages. It used the techniques of modern advertising. In many ways, it was part of the ‘society of the spectacle’, a product of that same society which it was attacking. But its message was radical and its title was bold and shocking – *Morire di Classe*.^7^

On the right-hand side of the rectangular cover, underneath the title, there was a long (unattributed) quote, which was from the Basaglias themselves. This citation was also reproduced inside the book in a slightly different version.^8^ It read as follows:

At the end of this process of de-humanization, the patient who has been entrusted to the psychiatric institution so that he can be cured, no longer exists: he has been eaten up and incorporated within the rules that determine his existence. He is a closed case. He has been labelled in an irreversible way, and he can no longer cancel those signs which mark him out as something which is not human, without any possibility of appeal. (Basaglia and Basaglia, 1969)

Right from its cover page, *Morire di Classe. La condizione manicomiale fotografata da Carla Cerati e Gianni Berengo Gardin* (edited by F. and F. Basaglia, 1969) presented itself as a different sort of book. It was not merely a collection of texts, debates and documents. Here, the images and photos themselves took centre stage. This was a design object, a political and sociological photobook, a book to be looked at (or looked away from) as much as read. As well as attempting to revolutionize mental health care, the Basaglias (along with Giulio Bollati who worked for the Turinese publisher Einaudi) were also attempting to transform the world of books and political campaigning. The appearance of *Morire di Classe* was a memorable moment in the history of the movement, and also in the history of publishing.

## The making of *Morire di Classe* (Basaglia and Basaglia, 1969)

That was the tough part of working in the mental institutions – managing to avoid portraying the illness, and instead showing the conditions in which the patients were living. In order not to inflict indignity, when Carla Cerati and I went to the mental institutions, we used to hold meetings to ask for the patients’ consent and to explain what we intended to do. And these so-called lunatics, who weren’t that mad at all, understood us perfectly and helped us. And then there was the extraordinary Basaglia. (Berengo Gardin, 2005a: 10)

*Morire di Classe* was put together in 1968–9 and published in May 1969. After the success of their collective book about their experiences in Gorizia, *L’istituzione negata* (1968), Basaglia and his wife met the photographer Carla Cerati through her husband Roberto, who was commercial director of Einaudi. The meeting took place in Milan in the spring of 1968 and Basaglia later wrote to Bollati; ‘I have spoken with Cerati’s wife about the possibility of an anti-institutional photobook. As you can see, I am not satisfied with what we have achieved so far’.^9^ According to Pitrelli, Basaglia originally wanted to produce a book that covered other institutions beyond asylums (and to some extent this is what he did) (Pitrelli, 2004: 86). It also seems that the photographers and Basaglia were well aware of the possible dangers in using images of patients (which explains the discussions and the requests for access discussed by Berengo Gardin). In addition, Basaglia did not want to be photographed with the patients (Cerati in Pitrelli, 2004: 85).^10^ He was worried about appearing to be paternalistic. Photos were taken of Basaglia, but they were not used in the book.

A plan was hatched for a new kind of book, using radical graphic design and photos from a series of asylums, which would be taken by Cerati and Berengo Gardin, whom Basaglia knew from his home town of Venice.^11^ Berengo Gardin later said that he was asked directly by Cerati to accompany her on her trips into a number of asylums. He agreed, he said, on the condition that he could also take photos alongside her.^12^ Carla Cerati later described the genesis of the book in this way: ‘I got in touch with him [Basaglia] through the publisher and I discovered that he wanted to find out how to create a photographic book about repressive institutions. I was very happy to participate in this project and Basaglia helped Berengo Gardin and me to get into a series of hospitals’.^13^

In the spring of 1968, nurses working with temporary contracts in the huge asylum in Colorno, just outside Parma, staged a demonstration^14^ about their job prospects (they were threatened with the sack) and also about the conditions inside the hospital itself. This was one of the most striking visual events of 1968, as a few nurses and demonstrators donned strait-jackets in the street. Berengo Gardin took photographs during the demonstration, some of which have appeared in later books linked to *Morire di Classe* (Brugnoli, 1998–9; Pivetta, 2012: 213–14). This demonstration is cited in numerous books and articles linked to the Basaglian movement and 1968 in general, and it has taken on semi-mythical status. It is also frequently moved in terms of time, to coincide with a photographic exhibition about asylums held in June 1968 in Parma. This seems a classic case of ‘mistaken’ remembering, where an event is shifted in order for it to ‘fit’ better with historical narratives.^15^

Cerati later remembered this event in an interview:

At the same time as the exhibition there was an important demonstration of nurses on short-term contracts, who marched through the streets of Parma wearing strait-jackets, in order to attract attention. These nurses told us that that one person, working alone, in the night, could not control up to seventy agitated patients without using strait-jackets. Their work was important in order to avoid using these forms of coercion. The nurses also told us that the patients were so used to seeing them as torturers that they would cower whenever they approached.^16^

Some of these photos from the nurses’ demonstration first appeared in an exhibition organized in part by a communist politician and ally of Basaglia, Mario Tommasini, in Parma in June 1968 for which Einaudi also provided publicity (see Fig. 4). The exhibition was called *La violenza istituzionalizzata* (Institutionalized violence) and was in part organized by the local Associazione per la lotta contro le malattie mentali (The Association for the struggle against mental illness), a newly-formed pressure group with branches in various cities across Italy which was working for the reform of mental health care.

The exhibition of photos of asylums opened in Parma on 1 June 1968 and lasted until 9 June (Brugnoli, 1998–9: 10, n.67). Basaglia wrote to the Einaudi editor Giulio Bollati about this event:

[Mario] Tommasini [a local Communist politician in Parma who was responsible for running the asylum and had been inspired by the Gorizia experiment] told me that they organized the exhibition in the courtyard where the street cleaners work, among rubbish bins and so on … Perhaps this is the right place for us to be, but I am a little worried about it … Let’s see what happens!^17^

This exhibition was the first public airing for many of the photos which would also appear in *Morire di Classe*. After Parma the exhibition moved onto Florence (see Fig. 4 for the original exhibition leaflets).

### The asylums photographed

The asylum photos that eventually appeared in the book came from three different hospitals – those in Colorno (near Parma), Gorizia and Florence – and were taken between April and October 1968.^18^ The book itself did not indicate which images were taken by which photographer, nor from which asylum they came. In fact, none of the photos in the book had captions. All of these asylums were undergoing some sort of change (and therefore were by no means the worst ‘total institutions’ in Italy). The two photographers also visited the asylum in Ferrara, but none of the photos from that trip were used in the book.^19^ Basaglia helped the photographers to gain access to the asylums in Gorizia and Parma. In Gorizia they were given the run of the hospital. In Florence, however, according to Cerati, the authorities were opposed to any photography at all, and the two photographers were allowed in only once, thanks to the help provided by two psychiatrists working there.

Cerati later recalled what happened there.

The experience in the Florence of asylum was the most traumatic for me … they told us that we were very lucky as they had just cleaned up as, usually, there was a about a metre of shit in the corridors … in any case it was horrible to see how the people there were suffering … ^20^

Cerati argued that:

During this work I felt the limits of the camera for the first time – it was impossible to capture in an effective way the obsessive repetition of gestures, the voices, the cries of the patients … but at the same time the impact of a still photo is much greater than that of those moving images which we view every day without noticing it on the small screen. (Bianchino, 2007: 162)

Forgacs (2014: 370–2) has since claimed that ‘photography itself was limited in terms of its impact in this context by its static nature and other features’. He concludes (p. 372) that ‘photography, even when accompanied by the kind of contextual material supplied in *Morire di Classe*, can provide a view of people with mental illness but it is an inadequate medium for providing an understanding of them, or even of the nature of their institutionalization’.

Things were certainly easier in Colorno thanks to the mediation of Tommasini and Basaglia, but it was not entirely straightforward once the photographers were inside. Not all the nurses in Parma were interested in reform (and this is also clear from the history of change in Colorno in the 1960s and 1970s), and it seems that some tried to seize the film used by Cerati and Berengo Gardin. According to Cerati, Berengo Gardin fooled them by handing over blank film.

**Figure 1.**
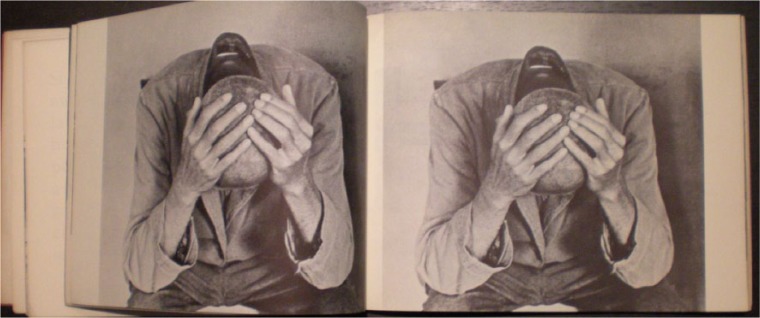
From *Morire di Classe*. Photo: Carla Cerati.

**Figure 2.**
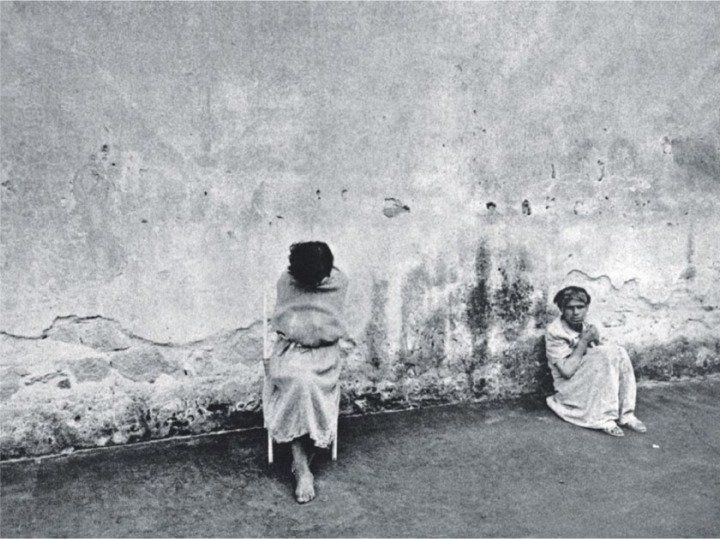
From *Morire di Classe*: Psychiatric Hospital, Florence, Italy; the cover image was taken from this photo. Photo: Gianni Berengo Gardin.

**Figure 3.**
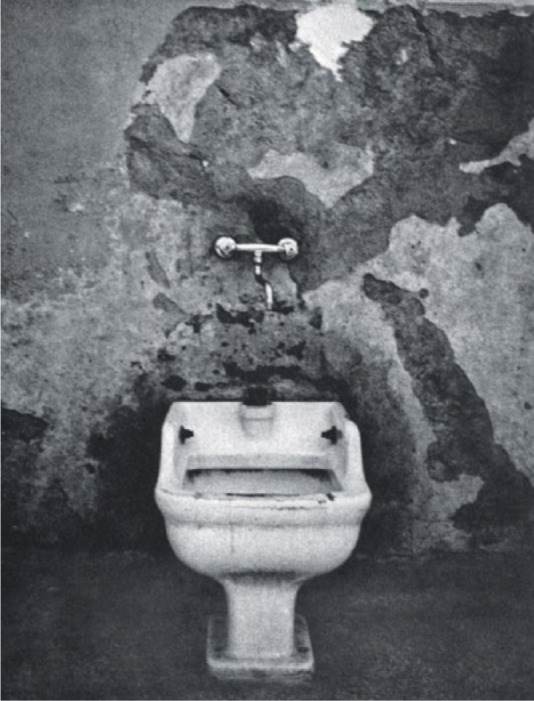
From *Morire di Classe*.

The photos taken were important for their context, for the environment in which the patients were to be found (with its bars, gates, concrete courtyards, huge walls) but above all because of the way that the patient’s bodies and faces seemed to betray signs of poverty and institutionalization, of pain, of suffering, of the rules and impositions imposed within the asylum (see Figs 1, 2, 3). Numerous other photos of various kinds were taken inside the asylums, especially Gorizia, including those that documented the transformations that had happened there. Berengo Gardin, for example, took shots of Basaglia himself, and of patient-led general meetings, but these were not published in *Morire di Classe*.^21^ That book did not aim to underline that any *change* was going on within asylums. Its goal was, in part, to shock. This was a political book, a violent statement of a state of affairs that, for its authors, had been allowed to continue for far too long. In this sense, at least, and certainly in the case of Gorizia, the book was also part-fiction, a construction, a tool of propaganda.

*Morire di Classe* was thus a little bit of a fake product, designed to work above all for reasons of politics and ‘the cause’. For example, the fact that a number of its images were taken in Gorizia in 1968 was somewhat strange, in a book of this kind. After all, this was an open hospital (in many ways the *most* open hospital in Europe at the time) without fences and gates and where nobody was tied up any more. Yet the photos were taken (or chosen) in such a way as if they were from an old-style *manicomio*. None of the photos showed the patients taking part in the activities associated with the Therapeutic Community there, or the general meetings that many other photographers came to Gorizia to document. This book depowered the patients, it put them back into a passive state, a state from which many (in Gorizia) had escaped. It ‘*re-victimized*’ them, in order to serve the needs of the movement. Of course, the photographers were also documenting or representing the institutional effects on patient’s bodies of years of internment in the asylum, but it was clear that the photos chosen from Gorizia were of a particular kind. They showed a past, not the Gorizian present (when they were taken).

By depicting patients as objects and not subjects, something that was profoundly anti-Basaglian, *Morire di Classe* presented a problematic vision of what was going on in Gorizia.^22^ It is perhaps for this reason that no images were chosen of the movement itself, or of meetings, and also that the places where the photos were taken were not indicated in the original volume. *Morire di Classe* was not a book that provided images of hope, or of change. Its message was blunt: *close these places down*.

### Post-production and publication

Franco Basaglia and Franca Ongaro chose the photos that were to be used, according to Cerati, although it is clear that Berengo Gardin also played a role during the production of the book, which was complicated by its unusual graphical form and shape. The editor, Giulio Bollati at Einaudi, who was a photographer himself and had a keen interest in the history of photography, played an important part.^23^ In Gorizia, it seems that the patients were paid for the photos taken, and decisions on the role of Cerati and Berengo Gardin were taken after meetings between patients and the photographers. It is not clear if similar meetings took place in Colorno and Florence, but it appears very unlikely that they did. There were no general patient meetings in Colorno or Florence or Ferrara at the time. In these cases, the photographers simply chose what to photograph, although they may have asked some individual patients for permission.

## The analysis of the asylum system and of society in *Morire di Classe*

The chronic [or incurable] nature of the patient is often implicit in the nature of the place where he or she is kept. But this nature does not depend in a direct way on their illnes. Recovery has a price, which is often very high, and this is therefore as much an economic and social question as it is a technical and scientific one. (Basaglia and Basaglia, 1969)

The photographs are both riveting and emotionally charged, causing reactions to oscillate between a sense of unwholesomeness and a sense of guilt. (Goffredo Fofi, quoted in Berengo Gardin, 2005a: 10).

*Morire di Classe* was a Basaglian book. Its analysis (both written and visual) contained all the elements of Basaglian thought as it was in 1969. In the introduction written by the Basaglias, all the key elements of Basaglian ideology were present, but no direct reference was made to the rest of the book (the photos and text extracts) in any specific way. That part of the volume was meant to speak for itself.

**Figure 4.**
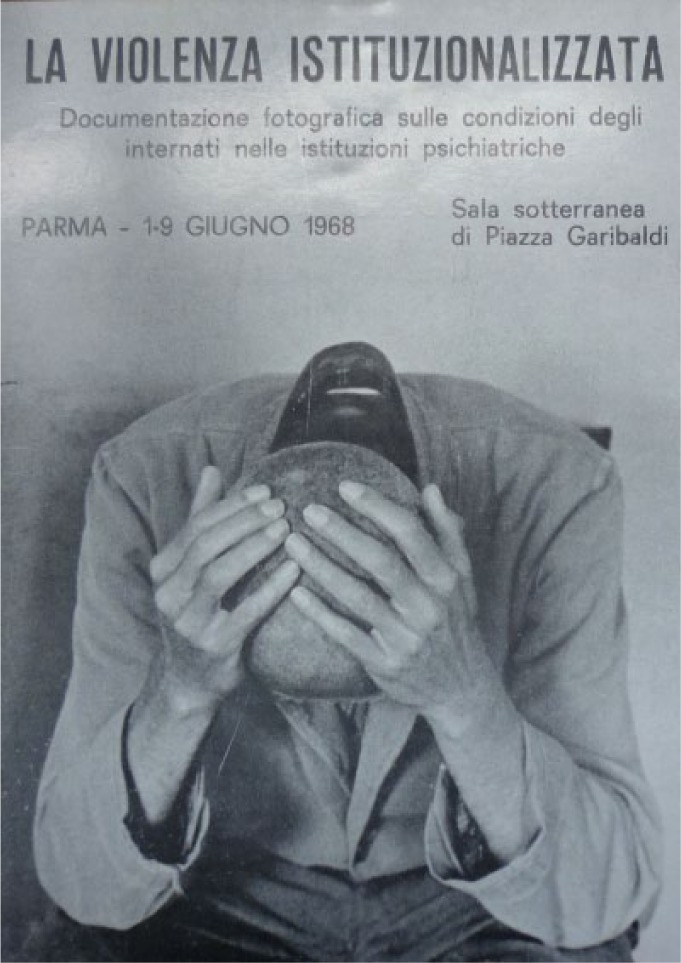
Leaflet from photographic exhibition, Parma, 1968 (Archivio Basaglia). Photo: Gianni Berengo Gardin.

Various aspects of Basaglian philosophy ran through the whole volume. First, there was the analysis of ‘total institutions’, through the citation of the work of Michel Foucault and of the socio-logist Erving Goffman and its illustration in images of various kinds. Goffman had published *Asylums* in 1961, a close study of the workings of a psychiatric hospital in the USA which developed into a key text with regard to the understanding of how psychiatric hospitals worked, and one which was later translated and published in Italian (Goffman, 1968), thanks to the Basaglias. They were particularly influenced by Goffman’s account of the ‘career of the mental patient’ and his use of the term ‘total institutions’. The narrative in *Morire di Classe* focused in part on the ‘architecture of containment’ – and thus there were shots of bars, keys, doors and patients tied up, and many images of patients within the architectural space of these institutions, often in passive, beaten down postures (see, for example, Figs 1, 2, 4). In their introduction, the Basaglias wrote of ‘keys, locks, bars, patients [which] are part of the furniture of the hospital which is run by nurses and doctors’ (Basaglia and Basaglia, 1969; Ongaro Basaglia, 1998: 9).^24^ The work of Frantz Fanon was also used – the asylum was described as a place of power, where the patient played the part of the ‘colonized’. Following Goffman, once the patient had become passive, a number, de-humanized, then their ‘career’ was complete. They had become ‘the perfect patient’.

Second, the effects of this violent (and often long-term) institutionalization could be seen on the bodies and faces of the inmates. At times, this was emphasized through the direct concentration camp-asylum comparison, underlined by a classic Basaglian citation from Primo Levi. Basaglian thought drew on Levi’s analysis of the concentration camp experience, and the reduction of people inside Auschwitz to ‘hollow’ or non-people. As the Basaglias wrote in the introduction: ‘the internee takes on the institution as part of their own body’ (1969; 1998: 8).

Third, there was an unambiguous social analysis of the asylum system (and of society itself). The world was divided into rich and poor, those who ‘had’, and those who ‘weren’t’, as Basaglia put it, using an Italian proverb. This was emphasized through images that were not of asylums at all, such as a bourgeois party with its rich and decadent guests, which were placed so as to contrast visually with pictures of patients. The stark and brutal title of the book was the boldest statement of all of this analysis: summarizing the social evaluation of the asylum that the Basaglias had adopted as their own. They wrote in their introduction that a patient inside the asylum was ‘at the same time, a poor person and an underdog who has no contractual power in order to resist the violence of the place, and who definitively comes under the control and power of a controlled … institution’ (1969; 1998: 10).^25^ *Morire di Classe* was also radical in other ways, in its style as well as its content. The book had no page numbers and individual photographs were not attributed to a particular author/photographer.^26^ It adopted an early form of ‘no copyright’, rejecting an authorial and ‘artistic’ view of the images in favour of a militant and political vision of the use of photography.^27^ It was a product of the counter-culture, in every sense.

The book also underlined a sense of protest over this state of affairs – it ‘denounced’ what was going on. It was angry, indignant, even. *All of this had to stop, now*. It was unacceptable. The introduction described the ‘overturning’ of Gorizia, and its limits. For the editors of the volume, ‘the beginning of a new form of therapy can only happen with the destruction of the asylum system, in order to create a new context where free forms of communication between doctors, nurses and patients can substitute – in the sense of support and protection – the walls, the bars and the violence’ (1969). What had the *équipe* done inside Gorizia? They had created a situation that had favoured ‘freedom of communication, and an attempt to destroy the rigid hierarchies of roles and eliminate the oppressive and punitive nature of the institution’. But social change was also needed. The movement could not confine itself to the *manicomo* (the Italian word for mad-house). It had to move on, beyond the asylum walls.

The first page of *Morire di Classe* (after the introduction) contains a photograph showing a bold paradox: a poster on a wall with an image of the heart transplant guru Christian Barnard, smiling with his hands folded in front of him and the slogan ‘*Le mani miracolose*’ [Miraculous hands]. Below the poster are three female asylum patients, all with heads bowed, two of them wrapped in long, blanket-like coats. The message is blunt. Modern medicine has done nothing to help *these* people: on the contrary.

*Morire di Classe* includes a series of quotes from a variety of eclectic works by the following: Levi – the much-used *If this is a Man* citation in Basaglian thought, which had also been used on the cover of the first collective book to come out of Gorizia, *Che cos’e la psichiatria?* (What is Psychiatry?; Basaglia, 1967) – Foucault, Goffman, Paul Nizan, Pirandello, the French psychiatrists Le Guillant and Bonnafé (with another quote which had already been used in *Che cos’e la psichiatria?*), Swift, Rainer Maria Rilke, Fanon, and others. The book also contains traces of voices of texts from patients themselves, such as a letter from an ex-patient, and some graffiti from a bathroom inside an asylum.^28^

The photos from Florence are the most shocking, the most raw and the most ‘real’ (although the places are not identified in the original book, we can work out which photos were from which institution from later texts and from the architecture shown in the photos). The environment is horrible: high concrete walls, isolated chairs, patients tied up in courtyards, fences, striped uniforms, strait-jackets, bodies splayed on the ground. This is an unreformed place, light years away from Gorizia (although not in the version seen in *Morire di Classe*). Some of the accompanying texts also underline the oppressive and at times absurd nature of the rules governing these institutions.

A series of images deal with the architecture of the total institution, with filthy toilets (Fig. 3) and baths, barred windows, rows of sinks, long corridors. Most of the patients shown are passive, sleeping, heads down and heads shaved. They are Goffmanesque ‘perfect patients’, beaten down by the system, reduced to Levi-like non-persons, to an animal state. In one photo a man is tied to a bed by his feet. It is relentless.

In some, however, the patients are more active, almost as if they are rebelling against the system in some way. Many seem to be suffering, in pain, crying out for help that would never arrive. One woman appears to be shouting, another grabs at the metal netting around the courtyard. Other photos from other places are more ‘human’, and some make direct contact with their subject matter, as with a patient who is staring straight into the camera. Not all the photos ‘de-humanize’ the patients, and some, in fact, achieve the opposite effect.

A celebrated series of four images, which have been much-used in subsequent years, depict a young woman with her eyes closed (in two of the images) and her head in her hands, shot through bars. In the next image, the same young woman is framed alone in the Gorizia courtyard, shot in a strange position, fixed in a silent scream. The final page of the book contains Frantz Fanon’s celebrated letter of resignation from the hospital where he had worked in Algeria – he was leaving in order to take up arms for the revolution. ‘My decision is’, he wrote, ‘at whatever cost, to accept no further responsibility under the false pretext that one cannot do otherwise.’ This letter had been cited in the appendix to *L’istituzione negata* in April 1968 in a well-known piece by Franco Basaglia, ‘Il problema della gestione’ (‘The question of management’; Basaglia, 1968). Four years later, in 1972, this same document would also inspire a celebrated collective resignation letter that would close down the whole Basaglian-Gorizian experience.

*Morire di Classe* was bang up to date and highly political. It was also a book about 1968 itself. In one (legendary) photo (taken by Berengo Gardin) a policeman seems about to hit a demonstrator/photographer.^29^ This image came from the 1968 demonstrations against the Biennale in Venice. Berengo Gardin had his hand damaged by the policeman just after taking this particular photograph. This was therefore a militant book, which sat well on the shelves of any self-respecting ‘68er’.

Occasionally, the contrasts drawn in the book are blunt and rhetorical. A shot of rich young people lounging around at a party, with a marble table and expensive paintings on the walls above them, is placed opposite a series of bodies/patients from an asylum, sprawled on the ground and all in uniform. One is in a strait-jacket. A real advert is included in order to show the dangers of integration and the cultural industry, which was using 1968 in order to sell its products. Ironically, the book itself, as we have pointed out, was clearly informed by modern advertising techniques.^30^ A stunning, powerful photo of a man, with a shaved head, head in hands (Fig. 1), is repeated three times. This image, taken by Carla Cerati in Colorno, had already become one of the symbols of the Basaglian struggle, appearing in campaign posters (e.g. Fig. 4) and in *I Giardini di Abele*, the documentary shot by Sergio Zavoli in Gorizia in 1968 and shown in 1969. This photo would be reproduced time and time again by the Basaglian movement in the years to come.^31^ Cosimo Schinaia later referred to it as ‘the symbol of marginalization in psychiatric hospitals’ (quoted in Parmiggiani, 2005a: 41).^32^ The head-in-hands image was already being used as propaganda for the movement before *Morire di Classe* was published (Babini, 2009: 262).^33^

The script and images in Sergio Zavoli’s important 1969 TV documentary shot in Gorizia – which brought Basaglia to the attention of ordinary Italians – were linked to and informed by the material in *Morire di Classe*. That documentary (*I Giardini di Abele*, 1969) also included a still of the Cerati head-in-hands photo. That book and film should be seen as complementary pieces, to be read and seen in parallel. Basaglia had long been interested in the use of visual media alongside texts, and had utilized cartoons and drawings by his childhood friend, the artist and author of graphic books Hugo Pratt, in earlier propaganda and publications.^34^ Basaglia would later involve artists and actors in work with patients and the public in Trieste, in the 1970s. The material used in *Morire di Classe* appeared in all kinds of different contexts, taking on a meaning which went way beyond that of photography itself.

## The legacy of *Morire di Classe*

With time, *Morire di Classe* became a classic work of Italian photography, cited in most histories of the discipline. There are gaps, however, between the reputation of the book and its real life as a ‘product’. Many analyses imply that is was a pure photobook, ignoring the way that it mixed text and images (the texts are rarely, if ever, cited). In addition, the volume is often discussed as if all the photos inside the book were of asylums, without reference to the wide selection of pictures and images and the particular layout and shape of the book. As it quickly went out of print, *Morire di Classe* was more cited than read. Moreover, it was not a publishing success when compared with the other books published by Einaudi and the Basaglias in the late 1960s and early 1970s.

*Morire di Classe* only went through two editions, and was out of print by the early 1980s. In total it sold around 11,000 copies.^35^ Today, somewhat ironically, it is a collector’s item. In 1998, on the 30th anniversary of 1968 (and of *L’istituzione negata*) as well as the 20th anniversary of the 1978 ‘Basaglia law’, which eventually closed down all asylums in Italy, a very different edition was put together; it was also given a new title, *Per non dimenticare. 1968. La realtà manicomiale di “Morire di classe*” (We must not forget. 1968. The reality of the asylums and *Morire di Classe*) (Ongaro Basaglia, 1998).

It is interesting to analyse how different this text was from the 1969 original. In the first instance, the choice of photos is different, with some new photos and many fewer ‘political’ images than in the 1969 edition. Secondly, the whole shape of the book has changed; it is a more conventional A4 size as opposed to the horizontal form of the original. Thirdly, many of the original texts have been removed. It is difficult to see this as a simple new edition. It is an entirely new publication.^36^

There are other points about *Per non dimenticare* which are worth noting. The book is edited by Franca Ongaro Basaglia alone (Franco Basaglia died in 1980), the cover has been changed (with the famous head-in-hands photo from Colorno there, taken by Carla Cerati), and the title is new and indulges in ‘memory rhetoric’. Thus the photo selection, order of the photos, layout and texts all differ from the original. There are also a number of photos that were not in the original volume. These include a series from the celebrated nurses’ demonstration in Parma in spring 1968, taken by Gianni Berengo Gardin. They are not from *within* a *manicomio*, despite the captions that accompany them. This mis-labelling has been repeated on numerous occasions in other publications.^37^ Despite the time that had passed, therefore, the propaganda aspects of these photos continued to take precedence over the ‘truth’ in terms of what they actually represented. Finally, the no-copyright nature of the original 1969 text had fallen by the wayside by 1998. All the photos in the 1998 edition have authors, and the places where they were taken are also identified. The 1998 text is didactic and explanatory; it might be argued that it is also far less effective.

### *The ‘effects’ of* Morire di Classe

‘Some forms of communication are socially important, such as that reportage about psychiatric hospitals, others are not’ (Carla Cerati, quoted in Biancino, 2007: 162).

As with the TV film shot in Gorizia *I Giardini di Abele* (1969), substantial claims are often made for the effects of *Morire di Classe* upon the Italian public, and its political ramifications. For example, Carla Cerati later said: ‘I am convinced that out photographs helped Franco Basaglia to get a law passed which closed down the psychiatric hospitals in Italy … the power of an image is very different from that of a written text. Words can be countered, images can’t!’^38^ Berengo Gardin has also made a similar argument: ‘Those places were unknown realities … it was important in terms of creating a sense of indignation, a movement which helped to get the 180 law passed.’^39^ In turn Franca Ongaro Basaglia wrote this in her ‘Prefazione’ in 1998: ‘Thirty years ago, these images caused a scandal and many people were angered by them.’ (Ongaro Basaglia, 1998: 6).^40^

And there is more. Giannichedda (2005: 16) recently argued that *Morire di Classe* is ‘a book … which had an enormous influence in terms of public opinion concerning the inhumanity of the conditions for patients inside asylums’. Schinaia (2005: 38) has written that that ‘The emotional impact of these photos on public opinion was huge’, and that the book ‘made a notable contribution to the building of a cultural basis for passing of the 180 law’. Meanwhile, Pitrelli (2004: 85) has argued that ‘a movement was born out of that volume which asked questions about how to represent mental illness and the people who suffered from it’. Finally, Curti (2013: 10) wrote recently that ‘*Morire di Classe* was to prove fundamental in the process that would lead to the passing, in 1978, of Law 180’.

Critics have also attributed great power to this text. For example, Carlo Arturo Quintavalle later wrote that ‘it was these photos, and those by Luciano d’Alessandro (1965–68), alongside the commitment of Basaglia and many others, which closed down the asylums, places of horrible segregation which had been ignored up to that point’ (Quintavalle, 2005; see also Zannier, 2012: 295–6, 303).^41^

*Morire di Classe*, it is claimed, played an important role in exposing an uncomfortable reality at the heart of Italian society which many Italians had tried to disregard, or simply were not aware of. As Manzoli (2004) has argued:

*Morire di Classe* became a manifesto for Basaglia and the doctors who rejected institutional psychiatry: in that photographic book, photography acted as an interpretation of social and scientific events but, most of all, it acted as a testimony, as communicative evidence of the horrifying conditions of mental hospitals and the need to abolish them.

However, is there any evidence to back up these kind of claims? Certainly, these were pictures of a kind which had rarely been seen before. Berengo Gardin has underlined the fact that by the end of the 1960s very few images had been seen of patients inside asylums. ‘That reportage … had an enormous impact? Yes, because nothing of this kind had ever been seen before. It certainly wasn’t easy to take photos inside asylums. In that period, in Italy, only Luciano d’Alessandro, a Neapolitan photographer, had done anything similar’.^42^

Many of these kinds of conclusions and analyses are circular. The asylums *were* abolished, there *was* a movement against the asylum system, *Morire di Classe was* published at the time when the movement was at its peak, and *therefore* it must have played a role in the success of the movement. However, the actual *effects* of this role, and the link between *Morire di Classe* and the battle for the 180 law are much more difficult to ascertain, or understand. It is not enough to simply state that these links existed. The book itself did not have a long print run, and many of the photos were used as propaganda in other contexts – as posters, on leaflets, on other book covers, and in the 1969 TV documentary that preceded the publication of *Morire di Classe*.

Historically, we can only understand the passing of the 180 law in 1978 in the context of a series of factors, events and cultural products which created the political space for the passing of that reform – including the creation of an Italian national health service, the financial costs of the asylum system, the role of television and a new generation of psychiatrists with different ideas from those of the past. We should be very careful when simply taking the supposed effects of *Morire di Classe* as read, or drawing a direct line between that book and the 180 law reform of 1978. In short, there is no evidence at all for many of the claims that are made about *Morire di Classe*.

## Conclusions: the many after-lives of *Morire di Classe*

Question: ‘If you wanted to photograph a madman now, would it be possible anymore?’ Carla Cerati, ‘I hope not’.^43^

The opening of a hospital and free communication can only work if the external world participates in the relationships which emerge. Free internal communication will remain artificial if there is not a constant dialogue between the inside and the outside … It is now important that the external world recognizes the psychiatric hospital as its own … in order to create a connection and a common interest between the institution which is changing and a society which desires the recovery of its people … Once the exclusionary nature of traditional psychiatric institutions has been made clear through the experimentation with new therapeutic possibilities, it is the external world which will determine how far it will be able to accept these new forms of communication. (Basaglia and Basaglia, 1969; Basaglia, 1982: 78)

*Morire di Classe* was a book that *did* prove central for the future careers and reputations of both Cerati and Berengo Gardin. Fusina later wrote: ‘It was a book that made Berengo Gardin famous among people who are not usually interested in photography’ (Fusina, 2005: 195). It also, along with the photos of d’Alessandro, started a major wave of ‘asylum photography’, which would lead to dozens of books and exhibitions running right through the 1970s and the 1980s. Berengo Gardin and Cerati had started a trend. They had created an entire genre.^44^

Since 1969, many of the photos from *Morire di Classe* have appeared time and again, in many different guises. For example, individual photos have been used on the covers of foreign editions of *L’istituzione negata*, and on posters and leaflets.^45^ Later Italian editions of this book also carried a cover image from *Morire di Classe*. These images thus became part of the movement, outside the specific context of the photobook itself, useful for the struggle against ‘total institutions’.

The idea of photographing asylums and those people who had been interned within them was widely used within the movement after 1969. In that same year Luciano d’Alessandro published a famous book based on a southern asylum, where he had been invited in by the radical psychiatrist Sergio Piro in 1965 in order to document the changes there (d’Alessandro, 1969; see also Piro, 2010: 64). In Perugia, also in 1969, the President of the Province, Ilvano Rasimelli, showed slides from the local asylum in the council chamber to try to convince his colleagues that the conditions inside the hospital were unacceptable. Later, a number of photographers would document the anti-asylum movement itself: Claudio Ermè and Gian Buttarini in Trieste and numerous other photographers elsewhere, right across Italy, including the celebrated French photographer Raymond Depardon (1984, 2014), who also made a full-length documentary film inside the Venetian asylum of San Clemente (*San Clemente*, 1982).^46^ *Morire di Classe* might not have changed very much in terms of the asylum system, but it certainly changed asylum photography and, more generally, the understanding of the role of photographers in Italy. Photography thus developed into a way of understanding both the ‘total institutions’ which were in place across Italy for much of the twentieth century, and the reformers, radicals and revolutionaries who attempted to change and destroy those institutions from the early 1960s onwards.

This article has analysed one of the most important texts from that period – the photobook *Morire di Classe*. I have argued that this book needs to be re-visited in terms of its real content and as a hybrid or mash-up of text (in different fonts and styles) and images, within a radical and original publishing design. I have also questioned some of the claims made for this book, and analysed in detail the philosophy expressed in the volume. Finally, I have outlined the impact of *Morire di Classe* and underlined the importance of seeing this book in the context of a wider cultural and historical movement, and as a percursor, not perhaps of a social, cultural or psychiatric revolution, but certainly as part of a revolution in photography itself, and its uses.

## Notes

1. See: http://www.photographers.it/articoli/carlacerati.htm (accessed 9.2.14). All translations are by the author of the present paper.

2. See also: Babini, 2009; Colucci and Di Vittorio, 2001; Donnelly, 1992; Fussinger, 2011; Scheper-Hughes and Lovell, 1987.

3. For the history of photography, asylums and mental illness, see: Ferro and Peloso, 1999; Forgacs, 2014: 314–415; Manzoli, 2004.

4. Basaglia and his wife had been instrumental in spreading Goffman’s ideas in Italy, with particular reference to his 1961 book *Asylums* (Goffman, 1961) part of which was published (and translated) by the Gorizia group in 1967 in their first collective book (Basaglia, 1967). In 1968 the entire text of *Asylums* came out with Einaudi in a translation by Franca Ongaro, Basaglia’s wife and close collaborator, and with an introduction by both Basaglias.

5. Forgacs (2014: 326) has also suggested an alternative translation of this title: ‘Dying of Class’.

6. This is the best analysis of *Morire di Classe*, and I would like to thank David Forgacs for letting me read a draft version of his chapter.

7. A PDF of the original edition of the book can now be found online, with two new introductory pieces added but without the cover images of the 1969 original; see: http://www.2001agsoc.it/materiale/sconfinamenti/Sconfinamenti.N14.pdf

8. Part of the citation (edited for *Morire di Classe*) is from Basaglia’s 1966 piece ‘Un problema di psichiatria istituzionale. L’esclusione come categoria socio-psichiatrica’, included in Basaglia, 1982: 309–28. The sentence beginning ‘The patient is a closed case’ was probably written specifically for *Morire di Classe*.

9. Letter dated 16.5.1968, Archivio Einaudi, Turin. Cerati had started off as a theatre set photographer in 1960. She published numerous books of photography including some extraordinary work in Milan during the 1960s and 1970s and later became a successful novelist.

10. There are various sources that appear to point to the fact that the photographers were aware of the dangers of exploiting or even humiliating the patients they were photographing; see also Pitrelli, 2004: 86–7. Cerati later said: ‘It was interesting to document such brutal places, but when you take the photos you feel that you are committing an act of violence in terms of the people in front of you’ (cited in Bianchino, 2007: 162–3). See also the comments from the commentary of Zavoli’s documentary, *I Giardini di Abele*, now in Zavoli, 2002: 365.

11. Berengo Gardin moved with his family to Venice from Rome after World War II. His father was originally from Venice and his family owned a glassware shop on the island of Murano. There was also a family shop in Calle Largo San Marco owned by his sisters. In 1962 he moved to Milan and came into contact with the rich *milieu* of writers, intellectuals and photographers in that city at the time. He is now one of the most celebrated Italian photographers of the post-war period. Berengo Gardin’s version of the birth of this book is slightly different from that of Cerati and Basaglia. He claims that Cerati had been due to visit the asylums for a magazine commission, and had asked him to accompany her as she did not want to go alone. Berengo Gardin (2005b: 423) has also stated that he convinced Basaglia to put together *Morire di Classe*,.

12. ‘Carla Cerati asked me to work on a reportage with her … she said that it was a difficult and complex job and she would prefer to work with another photographer. I accepted immediately.’; see: http://www.informatissimafotografia.it/art011.asp (accessed 3.3.13).

13. See: http://www.photographers.it/articoli/carlacerati.htm (accessed 1.3.13).

14. This demonstration took place in he centre of Parma on 7 April 1968. Nonetheless, many sources claim that this demonstration was explictly linked to the photographic exhibition held in Parma in June; Babini, 2009: 262; Ongaro Basaglia, 1991: 30–1. For more details on events in Parma, see: Braidi and Fontanesi, 1975: 25–30; Rossi, 2000: 181–3. Braidi and Fontanesi argue that the point of the demonstration was also to show that these nurses were needed to be able to remove the need to tie up patients. For an account of the demonstration, see also: ‘Insensibile esibizione nello sciopero degli infermieri dello psichiatrico’, *Il Resto del Carlino*, 7.4.1968. According to press reports in *La Gazzetta di Parma*, demonstrators ‘Dissensi sulla situazione all’ospedale psichiatrico’(‘pretended to be mentally ill patients’, 7.4.1968), and ‘Una dimostrazione di infermieri’ (8.4.1968).

15. The classic study of this kind of collective ‘error’ is Portelli, 1991.

16. See: http://www.photographers.it/articoli/carlacerati.htm (accessed 1.3.2013). This ‘mistake’ is repeated in *Gianni Berengo Gardin* (Fusina, 2005: 128, 130) with regard to photos reproduced on pages 128 and 130, which are described as ‘Mental institution, Parma 1968’ and ‘Mental institution (a patient in a strait-jacket), Parma 1968’. There are also similar issues with a famous photo of four men with keys dangling from their trousers. It appears as if this is a deliberately staged image, involving Basaglia and other doctors, but in *Gianni Berengo Gardin* the caption reads ‘Doctors with the keys to the cells of a mental institution, Parma’ (Fusina, 2005: 132); but see the alternative caption in *Il volto della follia* (Parmiggiani, 2005a: 80): ‘Franco Basaglia e altri psichiatri posano con i mazzi delle chiavi in uso nei manicomi, Parma, 1968’ (Franco Basaglia and other psychiatrists pose with bunches of keys from the asylum’). See also d’Amico, 1998: 98–9, 104–5.

17. Letter from Basaglia to Bollati, 23.5.1968. The inauguration of the exhibition was held in the evening.

18. Further details on these visits and various exhibitions, including the original publicity leaflets (e.g. Fig. 4), can be found in the Archivio Basaglia. The exhibition moved on to Florence straight after Parma.

19. Cerati has since said that they were not allowed to take photographs within Ferrara’s asylum, but an image from Ferrara taken by Berengo Gardin appears in Parmiggiani, 2005a: 210.

20. See: http://www.photographers.it/articoli/carlacerati.htm (accessed 1.3.13).

21. Other photos from the series have turned up in various publications over the years; see for example the photos of a meeting and of students in Gorizia, and of Franco Basaglia in Parmiggiani 2005a: 200–1. There are plans currently in place to publish a fuller set of Berengo Gardin’s asylum photos.

22. On this point, see also Forgacs, 2014: 372–3.

23. It is obvious that these photos are chosen with extreme care. It is therefore a little odd that Sandro Fusina (2005: 195) should write that *Morire di Classe* was ‘A book in which the photographs were deliberately printed at random’. The putting together of the book was not an easy process. Giulio Bollati wrote this in a letter to Basaglia (3.3.1969): ‘da Milano Berengo mi telefona tutti i giorni a mi crea non poche difficoltà avanzando le sue rivendicazioni di estetica fotografica. Temo che quando vedrà le prove stampate dirà che non vanno bene. Dovrò fare qualche acrobazia per portare in salvo l’edizione tra le opposte esigenza sue e di Giulio Einaudi’ (‘Berengo calls me up every day from Milan and he is causing a lot of problems with his claims about the aesthetics of photography. I think that when he sees the proofs he will not be pleased with them. I will have to be very creative if I am to find a balance between his requirements and those of Giulio Einaudi [the publisher]’); Archivio Einaudi.

24. These are the references indicated in citations below which give dates without authors. The 1969 book was unpaginated.

25. See also Forgacs, 2014: 324–8.

26. This was done later, in the re-new-edition of 1998 (see below).

27. However, this unusual choice clearly created tensions between the two photographers involved, and in the second edition of the book the individual images were attributed to their authors.

28. On the texts in the book and the layout, see also Forgacs, 2014: 324–35.

29. This photo was also included in *Morire di Classe*.

30. For the use of advertising techniques in the 1970s and protest movements, see Belpoliti, 2008.

31. The photo was promoted to the cover of a new but much-changed edition in 1998, *Per non dimenticare*. It also made the cover of an English language book about Basaglia called *Psychiatry in Transition* (Ramon and Giannichedda, 1988) where it was wrongly attributed to Berengo Gardin; it has also been used on album covers.

32. The photo itself is reproduced in Parmiggiani, 2005a: 213.

33. See also Tommasini: ‘La fotografia di un internato di Colorno (una testa china, coperta da due mani scarne, in atteggiamento disperato) diventò il manifesto, per l’Italia, della lotta al manicomio’ (‘The photograph of an inmate from Colorno (head bowed, covered with two thin hands, in an act of desperation) became the manifesto, in Italy, for the struggle against the asylum system’), cited in Ongaro Basaglia, 1991: 31; see also Pitrelli, 2004: 85. There is also an often used photo of Basaglia in front of the head in hands image, reproduced for example on the cover of the biography by Parmegiani and Zanetti, 2007 (the photo is also on p. 49 of the same book). See also Pivetta, 2012: 213–14. The photo was also seen in the *I Giardini di Abele* documentary (1969), first as a direct shot and then in posters on both walls of the office during Basaglia’s interview, and was used on the leaflet produced for the 1968 Parma exhibition (see Fig. 4).

34. The publisher Giulio Bollati played a key role in this book, as he had with *L’istituzione negata*. Bollati was interested in photography and its relationship with history. He was also a photographer himself; see: Bertelli and Bollati, 1979; Bollati, 1979, 2011: 128–85.

35. *Morire di Classe* (1969): 8092 sold (10,313 published); 2nd edn (1974): 2575 sold (3958 published); Franca and Franco Basaglia. Rendiconto al 31.12.1975, Turin, 12.7.1976, Archivio Einaudi.

36. In the introduction to her book, Giannichedda (2005: xxxi, n.59) writes: ‘The book was republished by the Edizioni Gruppo Abele in 1998 with the title *Per non dimenticare. 1968, la realtà manicomiale di morire di classe*.’

37. See: http://www.photographers.it/articoli/carlacerati.htm (accessed 1.3.13). See also the photo in Parmiggiani (2005a: 206), described as from the Ospedale psichiatrico provinciale, Parma, 1968.

38. See webpage given in Note 37.

39. ‘1969 Morire di classe. La fotografia per comunicare grandi temi’, Intervista a Gianni Berengo Gardin di Rossella Bigi; see: http://www.informatissimafotografia.it/art011.asp (accessed 3.3.13).

40. See also Slavich (1981): ‘These photographs played an important role in the process of insitutional denial which slowly, over twenty years, led to the overcoming of the asylum system in Italy.’

41. For the importance of the issue of asylums in the international context in 1968, see: Basaglia and Basaglia, 1998: 67–9; Weitzmann and Hobsbawm, 1998.

42. See webpage given in Note 39; see also d’Alessandro, 1981.

43. See webpage given in Note 37.

44. See, for example, Ernè, 2008.

45. The German edition (Basaglia, 1971) carried a Berengo Gardin photo from *Morire di Classe*. This edition also had a separate section with photos from *Morire di Classe*, merging the two books in some ways.

46. See also the section in the recent documentary *Journal de France*, 2012.

## References

[bibr1-0957154X14550136] Babini V (2009) Liberi tutti: manicomi e psichiatri in Italia: Una storia del novecento. Bologna: Il Mulino.

[bibr2-0957154X14550136] Basaglia F (ed.) (1967) Che cos’è la psichiatria? Parma: Amministrazione Provinciale di Parma.

[bibr3-0957154X14550136] Basaglia F (ed.) (1968) L’istituzione negata. Rapporto da un’ospedale psichiatrico. Turin: Einaudi.

[bibr4-0957154X14550136] Basaglia F (ed.) (1971) Die negierte Institution oder die Gemeinschaft der Augeschlossenen. Ein Experiment der psychiatrischen Klinik in Görz. Frankfurt: Surhkamp; translation of previous reference.

[bibr5-0957154X14550136] Basaglia F (1982) Scritti, II, 1968–1980. Dall’apertura del manicomio alla nuova legge sull’assistenza psichiatrica. Turin: Einaudi.

[bibr6-0957154X14550136] Basaglia F Basaglia F (eds) (1969) Morire di Classe. La condizione manicomiale fotografata da Carla Cerati e Gianni Berengo Gardin. Turin: Einaudi (unpaginated); 2nd edn 1974.

[bibr7-0957154X14550136] Belpoliti M (2008) La foto di Moro. Milan: Nottetempo.

[bibr8-0957154X14550136] Berengo Gardin G (2005a) A diary of Italy: half a century of photographs. Conversation with Goffredo Fofi. In: Fusina, 2005: 7–23.

[bibr9-0957154X14550136] Berengo Gardin G (2005b) ‘The Books’, interview by Fulvia Pagano. In: Fusina, 2005: 421–431.

[bibr10-0957154X14550136] Bertelli C Bollati G (eds) (1979) Annali della Storia d’Italia, 2 vols Turin: Einaudi.

[bibr11-0957154X14550136] Bianchino G (ed.) Carla Cerati. Milan: Skira.

[bibr12-0957154X14550136] Bollati G (1979) Note su fotografia e storia. In: Bertelli Bollati (1979) Vol. II, L’immagine fotografica (1845–1945), 3–55.

[bibr13-0957154X14550136] Bollati G (2011) L’Italiano. Il carattere nazionale come storia e come invenzione. Turin: Einaudi.

[bibr14-0957154X14550136] Braidi G Fontanesi B (1975) Se il barbone beve … cronache e documenti di una esperienza psichiatrica a Parma. Parma: Libreria Feltrinelli di Parma.

[bibr15-0957154X14550136] Brugnoli N et al (1998–9) Il Sessantotto a Parma. Nuovi movimenti politici e lotte sociali in una città dell’Emilia rossa. Annali Istituto Gramsci Emilia-Romagna (2–3): 197–230 Accessed (1 Mar. 2013) at: http://www.csmovimenti.org/PDF_approfondimenti/01_SessantottoParma.pdf.

[bibr16-0957154X14550136] Colucci M Di Vittorio P (2001) Franco Basaglia. Milan: Mondadori.

[bibr17-0957154X14550136] Curti D (ed.) (2013) Gianni Berengo Gardin. Stories of a Photographer. Venice: Marsilio.

[bibr18-0957154X14550136] d’Alessandro L (1969) Gli esclusi (fotoreportage da un’istituzione totale). Milan: Il Diaframma.

[bibr19-0957154X14550136] d’Alessandro L (1981) Tra la mia gente. Fotografie dal Mezzogiorno d’Italia 1952–1980. Bari: Dedalo libri.

[bibr20-0957154X14550136] d’Amico T (1998) Gli anni ribelli, 1968–1980. Rome: Riuniti.

[bibr21-0957154X14550136] Depardon R (1984) San Clemente. Paris: Centre National de la Photographie.

[bibr22-0957154X14550136] Depardon R (2014) Manicomio. Selected Madness: Secluded Madness. Göttingen: Steidl.

[bibr23-0957154X14550136] Donnelly M (1992) The Politics of Mental Health in Italy. London and New York: Routledge.

[bibr24-0957154X14550136] Ernè C (2008) Basaglia a Trieste. Cronaca del cambiamento. Viterbo: Nuovi Equlibri.

[bibr25-0957154X14550136] Ferro A Peloso P (1999) Iconografie del manicomio negli anni del superamento. Rivista sperimentale di freniatria. La rivista dei servizi di salute mentale 123(4): 277–282

[bibr26-0957154X14550136] Forgacs D (2014) Italy’s Margins: Social Exclusion and Nation Formation Since 1861. Cambridge: Cambridge UP.

[bibr27-0957154X14550136] Fusina F (2005) Gianni Berengo Gardin. Photographer. Turin: Einaudi.

[bibr28-0957154X14550136] Fussinger C (2011) ‘Therapeutic community’, psychiatry’s reformers and antipsychiatrists: reconsidering changes in the field of psychiatry after World War II. History of Psychiatry 22(2): 146–63. 21877384 10.1177/0957154X11399201

[bibr29-0957154X14550136] Giannichedda MG (2005) Introduzione. In: Franco Basaglia. L’utopia della realtà, ed. Basaglia FO Turin: Einaudi, vii–lii.

[bibr30-0957154X14550136] Gittins D (1998) Silences. The case of a psychiatric hospital. In: Chamberlain M Thompson P (eds) Narrative and Genre. London and New York: Routledge, 46–62.

[bibr31-0957154X14550136] Goffman E (1961) Asylums. Essays on the Social Situation of Mental Patients and Other Inmates. New York: Doubleday; 2nd edn published in 1974 (London: Pelican).

[bibr32-0957154X14550136] Goffman E (1968) Asylums. Le istituzioni totali: i meccanismi dell’esclusione e della violenza, translated by FO Basaglia. Turin: Einaudi.

[bibr33-0957154X14550136] Manzoli F (2004) Images of madness. The end of mental hospitals illustrated through photographs. Journal of Science Communication 3(2); accessed (24.11.14) at: http://jcom.sissa.it/archive/03/02/A030203/jcom0302%282004%29A03.pdf

[bibr34-0957154X14550136] Ongaro Basaglia F (1991) Vita e carriera di Mario Tommasini burocrate proprio scomodo narrate da lui medesimo. Rome: Riuniti.

[bibr35-0957154X14550136] Ongaro Basaglia F (ed.) (1998) Per non dimenticare. 1968. La realtà manicomiale di “Morire di classe”. Turin: Edizioni Gruppo Abele.

[bibr36-0957154X14550136] Parmegiani F Zanetti M (2007) Basaglia: una biografia. Trieste: Lint.

[bibr37-0957154X14550136] Parmiggiani S (ed) (2005a) Il volto della follia. Cent’anni di immagini del dolore. Milan: Skira.

[bibr38-0957154X14550136] Parmiggiani S (2005b) Volti e corpi di una ordinaria follia. In: Parmiggiani, 2005a: 11–24.

[bibr39-0957154X14550136] Piro S (2010) Quando ho i soldi mi compro un pianoforte. Conversazioni con un protagonista della psichiatria del ’900. Naples: Liguori editore.

[bibr40-0957154X14550136] Pitrelli N (2004) L’uomo che restituì la parola ai matti: Franco Basaglia, la comunicazione e la fine dei manicomi. Rome: Editori Riuniti.

[bibr41-0957154X14550136] Pivetta O (2012) Franco Basaglia. Il dottore dei matti. Milan: Baldini & Castoldi.

[bibr42-0957154X14550136] Portelli A (1991) The Death of Luigi Trastulli and other Stories: Form and Meaning in Oral History. New York: State University of New York Press.

[bibr43-0957154X14550136] Quintavalle AC (2005) Quando il Sessantotto è tutto in una foto. Corriere della Sera (27 settembre): 41.

[bibr44-0957154X14550136] Ramon S Giannichedda MG (1988) Psychiatry in Transition. The British and Italian Experiences. London: Pluto Press.

[bibr45-0957154X14550136] Rossi I (2000) “Pericolo a sé e agli altri edi di pubblico scandalo”. L’occupazione del Manicomio di Colorno: una lotta contro la violenza istituzionalizzata. In: Becchetti M, Parma dentro la rivolta. Tradizione e radicalità nelle lotte sociali e politiche di una città dell’Emilia rossa 1968–1969. Milan: Edizioni Punto Rosso, 175–217.

[bibr46-0957154X14550136] Scheper-Hughes N Lovell A (1987) Psychiatry Inside-Out: Selected Writings of Franco Basaglia. New York: Columbia University Press.

[bibr47-0957154X14550136] Schinaia C (2005) Chiaroscuri. Sui rapporti tra fotografia e psichiatria. In: Parmiggiani, 2005a: 33–48.

[bibr48-0957154X14550136] Slavich A (1981) Morire di classe. In: Crepet P Giannichedda MG (eds) Inventario di una psichiatria. Milan: Electa, 53.

[bibr49-0957154X14550136] Weitzmann M Hobsbawm EJ (1998) 1968. Magnum Throughout the World. Paris: Hazan.

[bibr50-0957154X14550136] Zannier I (2012) Storia della fotografia italiana. Tomo 1, dalle origini agli anni ’50. Castel Maggiore: Editrice Quinlan.

[bibr51-0957154X14550136] Zavoli S (2002) Diario di un cronista. Lungo viaggio nella memoria. Rome: Rai-Eri.

